# Generalized morphea following COVID‐19 vaccine: Case report and literature review

**DOI:** 10.1002/ccr3.7133

**Published:** 2023-03-30

**Authors:** Raid Alhayaza, Ghada Alhayaza, Abdullah Algarni, Ahmed Alhumidi, Ruaa AlHarithy

**Affiliations:** ^1^ College of Medicine Alfaisal University Riyadh Saudi Arabia; ^2^ Department of Dermatology and Dermatologic Surgery Prince Sultan Military Medical City Riyadh Saudi Arabia; ^3^ Department of Dermatology King Saud Medical City Riyadh Saudi Arabia; ^4^ Department of Pathology King Saud University Riyadh Saudi Arabia; ^5^ College of Medicine Princess Nourah Bint Abdulrahman University Riyadh Saudi Arabia; ^6^ Department of Dermatology Security Forces Hospital Riyadh Saudi Arabia

**Keywords:** case report, COVID‐19 vaccine, generalized morphea, morphea, Pfizer‐BioNTech, SARS‐CoV‐2

## Abstract

Physicians should be vigilant for COVID‐19 vaccine side effects and investigate any associated cutaneous manifestations. This will ultimately facilitate better understanding and recognition of various skin reactions related to the vaccine.

## 1 INTRODUCTION

Adverse muco‐cutaneous manifestations were increasingly observed following COVID‐19 vaccinations, highlighting that such unfortunate cutaneous reactions are not only secondary to SARS‐CoV‐2 infection but rather can be related to COVID‐19 vaccines.^1^, ^2^ Particularly, Type 1 hypersensitivity reactions such as angioedema, anaphylaxis, and urticaria. Also, Type 4 hypersensitivity reactions such as erythema multiforme‐like rashes, morbilliform, and inflammatory markers at the injection site.^1^, ^2^ Furthermore, there are other cutaneous manifestations that were reported in the literature, including cutaneous vasculitis, pityriasis rosea‐like reactions, functional angiopathies, lichenoid drug eruptions, and herpes zoster reactivations.^2^, ^3^, ^4^, ^5^

The development of morphea secondary to vaccination is rare and the reported cases appear to be in predisposed patients, the majority of whom were children. Although the main mechanism behind it is still unknown, it was hypothesized to be due to antigenic cross‐reactivity between human tissues and vaccine spike proteins. This could subsequently result in immune‐mediated diseases like generalized morphea, where memory cells and antibodies are produced.^6^, ^7^ Additionally, polyarthritis, myocarditis–pericarditis, autoimmune disease flares, and delayed cutaneous reactions are further examples of immune‐related diseases and autoimmune activities that might result following the aforementioned interaction.^6^

Physicians should be vigilant for COVID‐19 vaccine side effects and investigate any associated cutaneous manifestations. This will ultimately facilitate and establish a better understanding and recognition of various skin reactions related to the vaccine. Currently there are few reported cases in the literature of morphea secondary to COVID‐19 infection.^1^, ^3^ On the other hand, there are much fewer articles describing morphea secondary to COVID‐19 vaccines itself^6^, ^7^, ^8^, ^9^, ^10^ (Table 1). Herein, we present one of the rare cases of new‐onset generalized morphea following COVID‐19 vaccine. In addition, we shed light on similar reported cases.

**TABLE 1:** All the reported cases in the literature of patients developing morphea following COVID‐19 vaccine including this case report. The table compares all the cases based on several factors such as age, gender, COVID‐19 vaccine type, interval between vaccination and symptoms, and other variables.

Case number and author	Gender	Age	Past medical/allergic history	Ongoing therapies	COVID‐19 vaccine name	First or second dose	Interval between vaccination & symptoms	Skin manifestation following vaccination	Number and diameter of the lesions	Body area and symptom	Labs, antibodies and DIF	Histopathologic findings	Treatment	Outcome
1 Alhayaza et al. (this case)	F	63	Diabetes & hypertension	Insulin and metformin	Corminaty® Pfizer‐BioNTech BNT162b2	Second dose	15 days	Erythematous and sclerotic plaques all over the body	*n* = ≥ 13 ø = 3–17.5 cm	Chest, axilla, abdomen, back	ESR:52 Complete rheumatology panel was negative.	Thickened collagen bundles in dermis with focal interstitial lymphocytes	Clobetasol propionate and MTX 15 mg/week for 3 months. Folic acid 5 mg/week. Petrolatum	N/A
2 Paolino et al.^8^	F	61	Negative	None	Corminaty® Pfizer‐BioNTech BNT162b2	First and second dose	15 days from both first and second dose	Generalized morphea, characterized by ≥10 plaques and ≥5 cm in diameter. No scleroderma.	*n* = ≥ 10 ø = 5–12 cm	Abdomen, back, lower limbs	ANA 1:160 Homogeneous	Dermal sclerosis consistent with morphea	Clobetasol 0.05% cream and MTX 7.5 mg/week. Because hepatotoxicity MTX has been replaced with mycophenolate	Good improvement
3 Paolino et al.^8^	F	52	Eosinophilic fasciitis	Abatacept and methotrexate from 2018	Corminaty® Pfizer‐BioNTech BNT162b2	Second dose	7 days	Generalized morphea, characterized by ≥5 plaques and ≥5 cm in diameter. No scleroderma.	*n* = 5 ø = 5–6 cm	Abdomen, chest, upper limbs	None	Dermal sclerosis consistent with morphea	Methotrexate 7.5 mg/week	Good improvement
4 Paolino et al.^8^	M	64	Negative	None	Vaxzevria® AstraZeneca ChAdOx1n Cov‐19	First dose	20 days	Generalized morphea, involving ≥2 anatomical regions. No scleroderma	*n* = ≥ 7 ø = 7 cm	Upper limbs, abdomen	ANA 1:160 Homogeneous	Dermal sclerosis consistent with morphea	Tacrolimus 0.1% cream	Improvement
5 Paolino et al.^8^	F	73	Atrio‐ventricular block	Pacemaker	Corminaty® Pfizer‐BioNTech BNT162b2	Second dose	20 days	Generalized morphea, involving ≥2 anatomical regions. No scleroderma.	*n* = > 5 ø = 5–6 cm	Lower limbs, abdomen	ANA 1:320 Homogeneous DIF: IgG‐, IgA‐, IgM‐, C3‐, C1q ‐, fibrinogen‐	Dermal sclerosis consistent with morphea	Tacrolimus 0.1% cream	Good improvement
6 Antoñanzas et al.^2^	F	45	Negative	None	Spikevax® Moderna mRNA‐1273	First dose	14 days	Brownish, patchy oval indurated lesions on her back	N/A	Back of the patient	Negative autoantibodies	A normal epidermis with thick collagen bundles	Betamethasone and topical calcipotriol	Great improvement
7 Antoñanzas et al.^2^	F	52	Negative	None	Corminaty® Pfizer‐ BioNTech BNT162b2	Second dose	6 weeks	Brownish patches on the abdomen and thighs	N/A	Abdomen and thighs	ANA and ANCA autoantibodies were negative	Unaltered epidermis with thickening of the collagen bundles of the reticular dermis	Topical corticosteroids and oral methotrexate 15 mg/m^2^/week	Lesions were seen to regress following methotrexate initiation
8 Metin et al.^3^	F	55	Negative	None	Corminaty® Pfizer‐BioNTech BNT162b2	Second dose	4 weeks	Red rash on the left breast. It started as a small red patch and it gradually spread over the armpit and breast	*n* = N/A ø = there was an ill‐defined indurated plaque measuring 20 × 10 cm	Armpit and breast. Nonpruritic	Post‐immuno‐histochemical analysis with SARS‐CoV‐2 anti‐spike protein antibody.	Epidermis with acanthosis, mild hyperkeratosis and a dermis with perivascular interstitial mononuclear cell infiltrates and coarse collagen fibers	Topical clobetasol propionate pomade and calcipotriol pomade	Lesions were seen to be regressed
9 Aryanian et al.^4^	F	70	Negative	None	Vaxzevria® AstraZeneca ChAdOx1n Cov‐19	First dose	2 days	Diffuse skin eruptions and maculopapular rash extending from arms to all body surface	N/A	Arms, and extending to all the body surface. including intertriginous areas	Complete rheumatology panel and tumor markers were negative	Sclerodermoid changes	Methotrexate and topical corticosteroids	N/A
10 Shakoei et al.	F	25	Hyperlipidemia, diabetes	Atorvastatin, metformin	Vaxzevria® AstraZeneca ChAdOx1n Cov‐19	First dose	10 days	Generalized sclerotic lesions	Not measured	Pruritus	ESR:51 CRP:5	N/A	Prednisolone, methotrexate	Mild improvement

## 2 CASE REPORT

**FIGURE 1:**
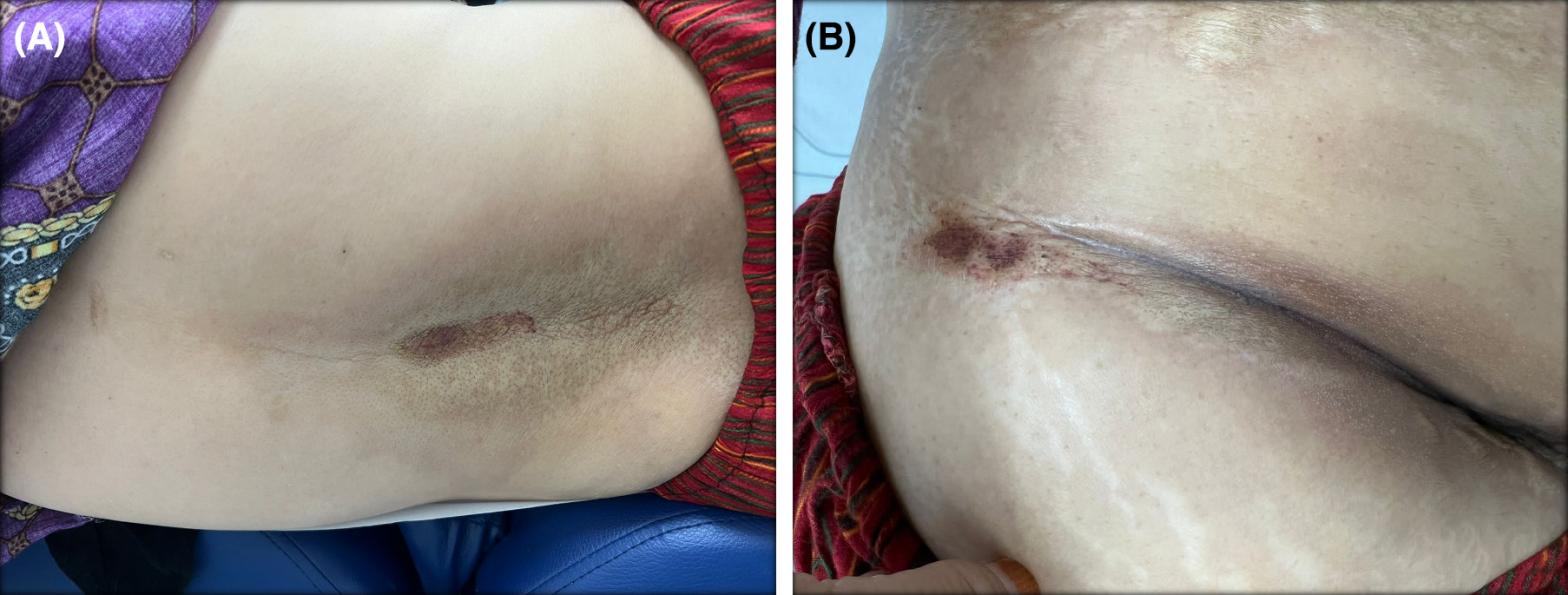
(A) The largest lesion out of the patient's generalized morphea, located on the back and it measures 17.5 × 8 cm. (B) Morphic lesion located in the infra‐abdominal fold.

**FIGURE 2:**
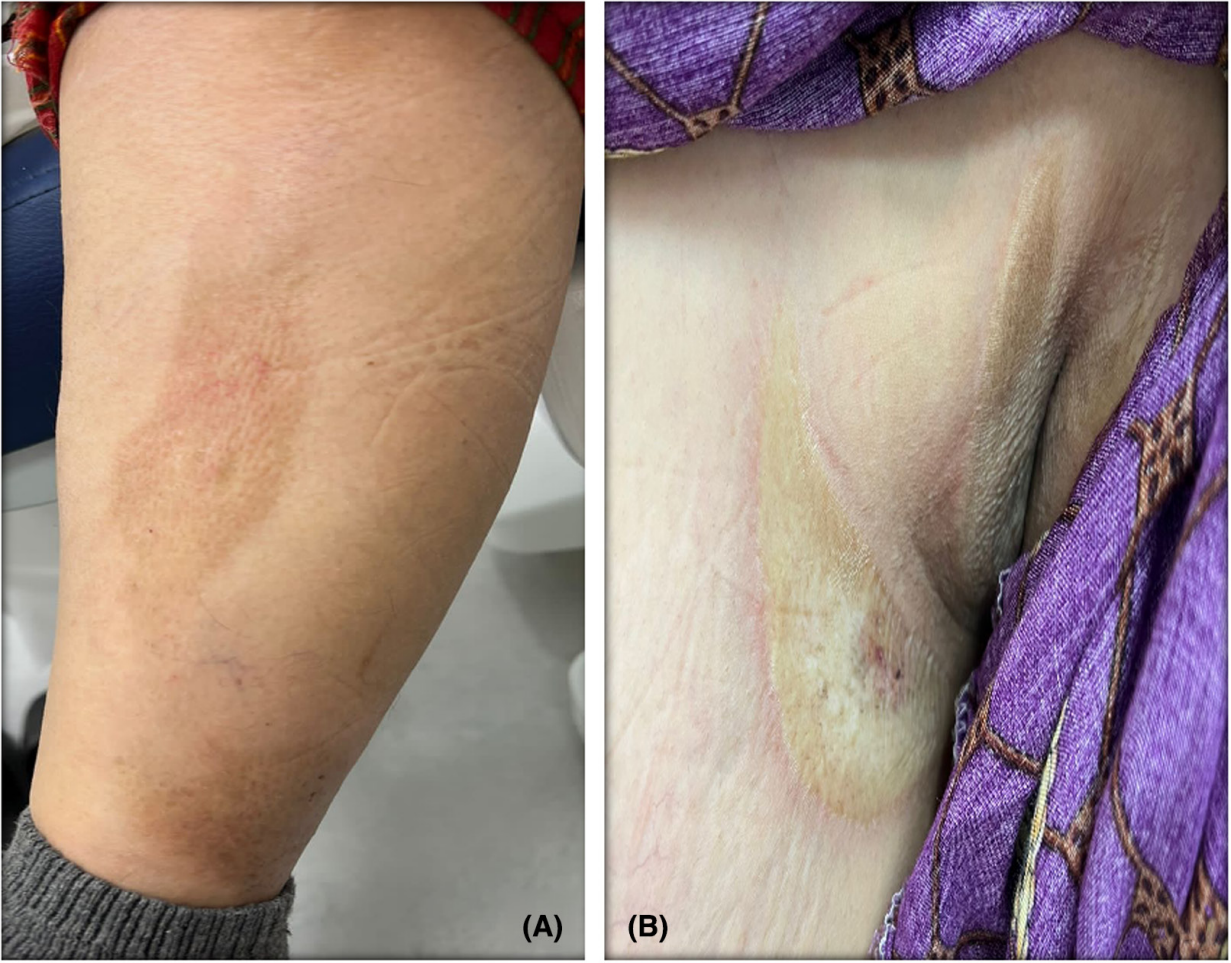
(A) Morphea in the patient's lower limb. (B) Morphea in the left axilla.

Our patient is a 63‐year‐old Saudi female, known case of hypertension and diabetes mellitus with uncontrolled blood sugar levels, on insulin and metformin. She presented to our clinic with erythematous and hyperpigmented sclerotic plaques over trunk and extremities. Interestingly, the patient noticed the lesions starting to appear 2 weeks after receiving her second dose of Pfizer‐BioNTech COVID‐19 vaccine 1 year ago. It first started as a solitary lesion over her right shoulder. Then, one to two new lesions were appearing every month. She denied any history of myalgia, arthralgia, or malaise. She had no personal or family history of autoimmune or dermatologic diseases. On physical examination, multiple scattered hyperpigmented atrophic hard plaques were seen over her left upper chest, axillae, abdomen, and back (Figures 1 and 2). A total of 13 lesions varying in size from 3 to 17.5 cm in diameter were observed. The largest one was located on the back as a centered linear hyperpigmented atrophic plaque with erythematous verrucous surface measuring (17.5 × 8 cm) (Figure 1a).

**FIGURE 3:**
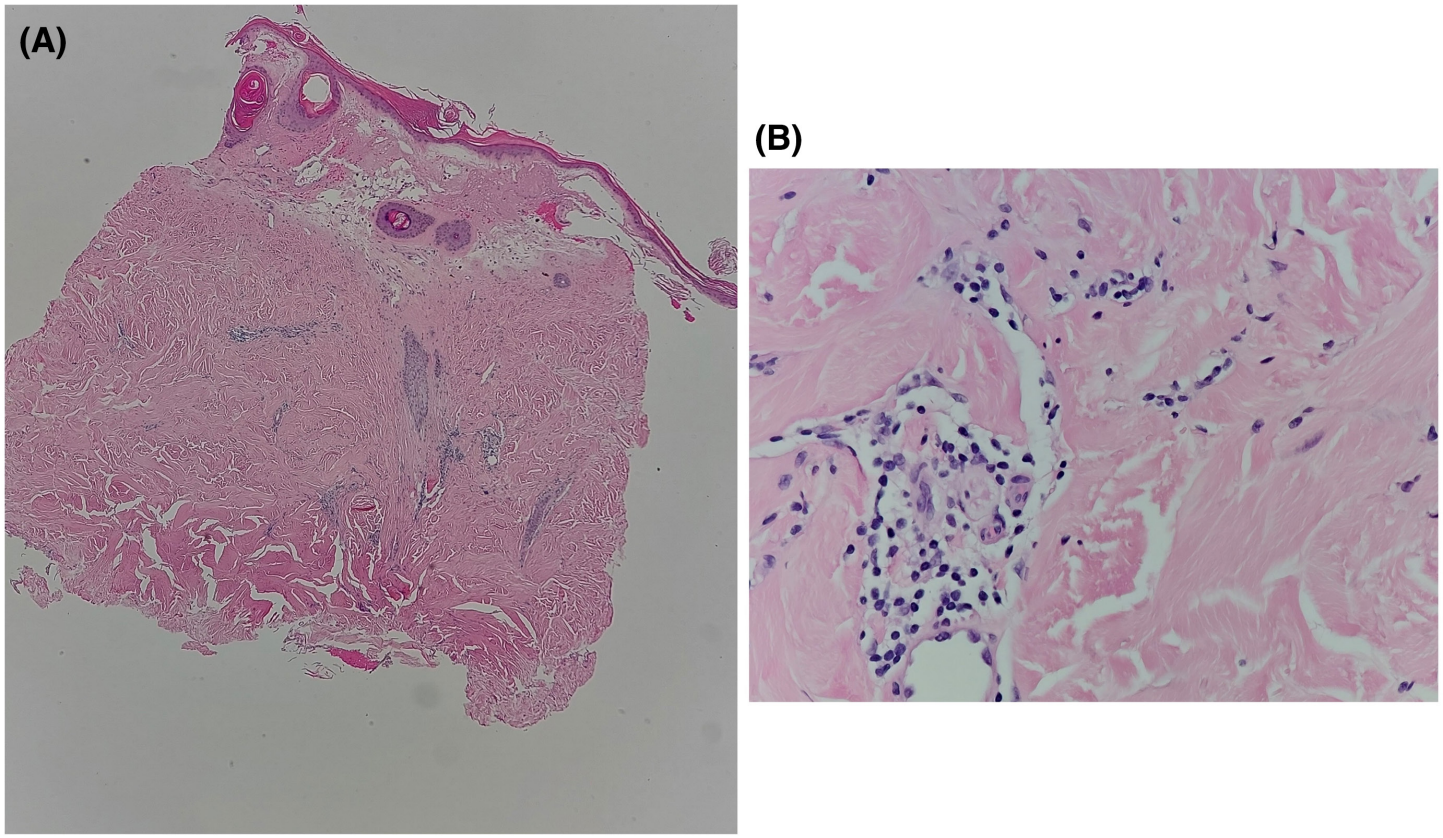
Histopathology (A) Photomicrograph shows square shape punch biopsy with hyperkeratosis, epidermal atrophy, papillary dermal edema, and thickened collagen bundles (H/E stain, original magnification × 40). (B) A higher power reveals thickened collagen bundles and interstitial lymphocytic infiltrate (H/E stain, original magnification × 400).

**FIGURE 4:**
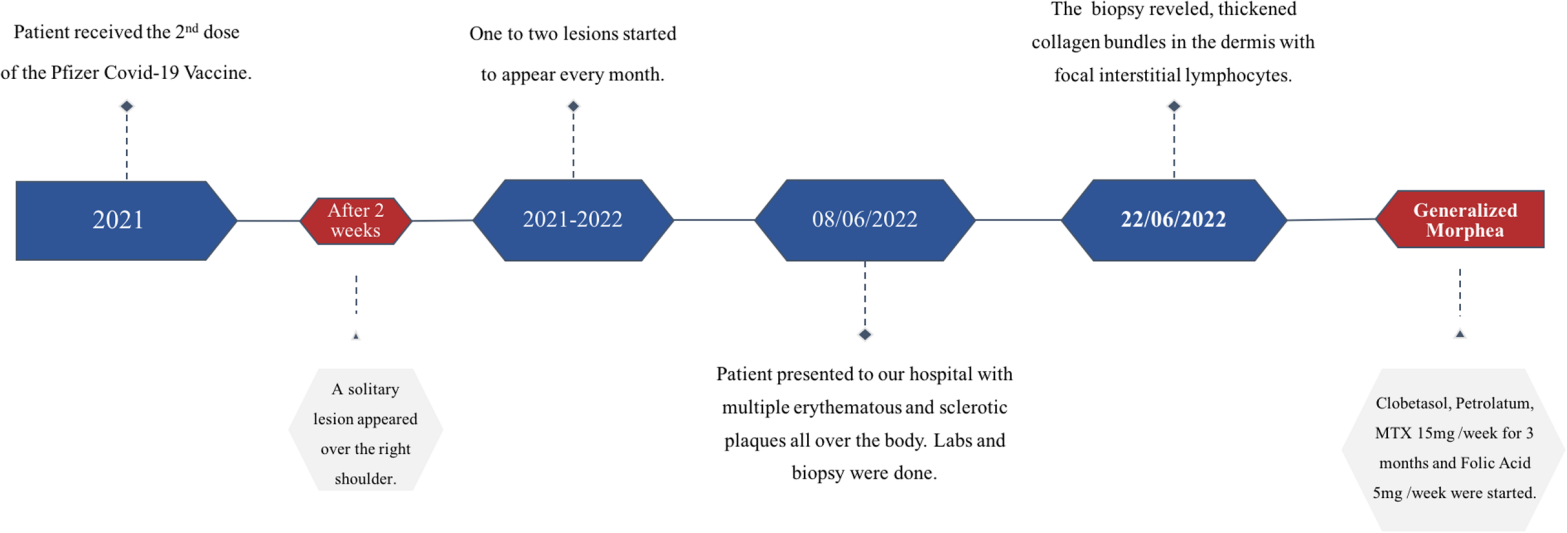
Represent the timeline of events in our case. Starting with the patient's history of developing solitary lesion shortly after receiving the second dose of Pfizer Covid‐19 vaccine, and ending with the diagnosis of generalized morphea and its management.

Therefore, the following were considered as a possible diagnosis: necrobiosis lipoidica and morphea. Laboratory tests were requested and all of which were within the normal range such as CBC, LFT, RFT, electrolytes, and CRP except for an increase in her ESR:52. Lastly, her autoimmune profile (ANA, anti‐DNA, anti‐SSA, anti‐SSB, anti‐SM, anti‐Jo1, anti‐SCL70, anti‐RNP, anti‐centromere) was unremarkable. Subsequently, a 4‐mm punch biopsy was done, which revealed thickened collagen bundles in the dermis with focal interstitial lymphocytes (Figure 3). The patient was prescribed methotrexate 15 mg weekly and her response to the treatment will be followed up and assessed. The timeline highlighting our patient's presentation is summarized in Figure 4.

## 3 DISCUSSION

Vaccinations enhanced global health and eradicated fatal infectious diseases such as smallpox. There are concerns about vaccine‐induced adverse events; however, its reported frequency in the most common vaccines ranges between 4.8 and 83.0 per 100,000 doses.^11^, ^12^ Vaccination may lead to the appearance of some dermatoses. Antoñanzas et al. have summarized distinct examples such as granuloma annulare development within a close temporal period following antitetanic vaccine administration.^7^ Additionally, patients developed bullous pemphigoid, mast cell tumors, and lichen planus after receiving hepatitis B vaccination. In those patients, the skin manifestations appeared only a few weeks after they received their vaccines. Lesions also developed exactly at the vaccine injection site, which possibly suggests a causal relationship.^7^

A total of nine patient presentations of morphea post‐COVID‐19 vaccination were reported in the literature.^6^, ^7^, ^8^, ^9^, ^10^ Four of which were following Pfizer‐BioNTech such as our case, three were post AstraZeneca vaccine, and one was post‐Moderna vaccine.^6^, ^7^, ^8^, ^9^, ^10^ Interestingly, all of the reported patients were female except one. The time interval before onset of lesions ranged between 2 and 15 days post‐vaccine, and half of the patients reported onset after the first dose while the other half after the second dose of the vaccine. Table 1 summarizes all the reported cases including our case report, comparing all reported variables. COVID‐19 vaccines can rarely cause immune dysregulation, which could consequently worsen an underlying condition or result in a new‐onset of a skin manifestation.^10^

Our patient developed generalized morphea 2 weeks after receiving her second dose of Pfizer‐BioNTech COVID‐19 vaccine. Using the WHO‐UMC system for standardized causality assessment our patient's reaction falls under likely.^13^ Moreover, morphea is also known as localized scleroderma, and is classified as an idiopathic, inflammatory, and sclerosing condition of the skin and subcutaneous tissue. It has been suggested that immune dysregulation plays a pivotal role in its pathogenesis along with genetic and environmental factors.^6^ Usually the clinical presentation of morphea lesions are atrophic or indurated brownish plaques that develop at the injection site and can become generalized and might even lead to functional impairment.^6^

Gambichler et al. concluded that due to the molecular mimicry between SARS‐CoV‐2 vaccine and human proteins, activation of autoreactive lymphocytes and recruitment of chemokines and cytokines leads to more widespread phenotype of autoimmune diseases.^2^

## 4 CONCLUSION

The pathogenesis of morphea is multifactorial. Immune dysregulation, genetic predisposition and environmental factors all play a role in disease activity. Dermatologic conditions related to COVID‐19 vaccines could range from mild to severe forms. Thus, it needs to be carefully monitored and should not be treated lightly. Further studies are needed to investigate and highlight the mechanism by which COVID‐19 vaccine results in morphea in certain people.

## AUTHOR CONTRIBUTIONS

**Raid Alhayaza:** Methodology; project administration; visualization; writing – original draft; writing – review and editing. **Ghada Alhayaza:** Conceptualization; project administration; supervision; writing – original draft; writing – review and editing. **Abdullah AlGarni:** Project administration; writing – review and editing. **Ahmed Alhumidi:** Investigation; writing – review and editing. **Ruaa AlHarithy:** Conceptualization; data curation; methodology; project administration; writing – review and editing.

## FUNDING INFORMATION

None.

## CONFLICT OF INTEREST STATEMENT

None.

## CONSENT

Written informed consent was obtained from the patient to publish this report in accordance with the journal's patient consent policy.

## Data Availability

The data used to support this study are included within the article and are available from the corresponding author upon request.
